# Robust and Scalable Learning of Complex Intrinsic Dataset Geometry via ElPiGraph

**DOI:** 10.3390/e22030296

**Published:** 2020-03-04

**Authors:** Luca Albergante, Evgeny Mirkes, Jonathan Bac, Huidong Chen, Alexis Martin, Louis Faure, Emmanuel Barillot, Luca Pinello, Alexander Gorban, Andrei Zinovyev

**Affiliations:** 1 Institut Curie, PSL Research University, 75005 Paris, France; jonathan.bac@cri-paris.org (J.B.); alexis.martin1@edu.ece.fr (A.M.); louis.faure@meduniwien.ac.at (L.F.); emmanuel.barillot@curie.fr (E.B.); 2 INSERM U900, 75248 Paris, France; 3 CBIO-Centre for Computational Biology, Mines ParisTech, PSL Research University, 75006 Paris, France; 4 Sensyne Health, Oxford OX4 4GE, UK; 5 Center for Mathematical Modeling, University of Leicester, Leicester LE1 7RH, UK; emmirkes@gmail.com (E.M.); a.n.gorban@leicester.ac.uk (A.G.); 6 Lobachevsky University, 603000 Nizhny Novgorod, Russia; 7 Centre de Recherches Interdisciplinaires, Université de Paris, F-75000 Paris, France; 8 Molecular Pathology Unit & Cancer Center, Massachusetts General Hospital Research Institute and Harvard Medical School, Boston, MA 02114, USA; huidong.chen@mgh.harvard.edu (H.C.); lpinello@mgh.harvard.edu (L.P.); 9 Broad Institute of MIT and Harvard, Cambridge, MA 02142, USA; 10 ECE Paris, F-75015 Paris, France; 11 Center for Brain Research, Medical University of Vienna, 22180 Vienna, Austria

**Keywords:** data approximation, principal graphs, principal trees, topological grammars, software

## Abstract

Multidimensional datapoint clouds representing large datasets are frequently characterized by non-trivial low-dimensional geometry and topology which can be recovered by unsupervised machine learning approaches, in particular, by principal graphs. Principal graphs approximate the multivariate data by a graph injected into the data space with some constraints imposed on the node mapping. Here we present ElPiGraph, a scalable and robust method for constructing principal graphs. ElPiGraph exploits and further develops the concept of elastic energy, the topological graph grammar approach, and a gradient descent-like optimization of the graph topology. The method is able to withstand high levels of noise and is capable of approximating data point clouds via principal graph ensembles. This strategy can be used to estimate the statistical significance of complex data features and to summarize them into a single consensus principal graph. ElPiGraph deals efficiently with large datasets in various fields such as biology, where it can be used for example with single-cell transcriptomic or epigenomic datasets to infer gene expression dynamics and recover differentiation landscapes.

## 1. Introduction

One of the major trends in modern machine learning is the increasing use of ideas borrowed from multi-dimensional and information geometry. Thus, geometric data analysis (GDA) approaches treats datasets of various nature (multi-modal measurements, images, graph embeddings) as clouds of points in multi-dimensional space equipped with appropriate distance or similarity measures [1]. Many classical methods of data analysis starting from principal component analysis, correspondence analysis, K-means clustering, and their generalizations to non-Euclidean metrics, or non-linear objects—such as Hastie’s principal curves—can be considered part of GDA [2]. Topological data analysis (TDA) focuses on extracting persistent homologies of simplicial complexes derived from a datapoint cloud and also exploits data space geometry [3]. Information geometry understood broadly can be defined as the field that applies geometrical approach to study spaces of statistical models, and the relations between these models and the data [4].

Local and global intrinsic dimensionalities are important geometrical characteristics of multi-dimensional data point clouds [5,6]. Large real-life datasets frequently contain both low-dimensional and essentially high-dimensional components. One of the useful concepts in modern data analysis is the complementarity principle formulated in [7]: the data space can be split into a low volume (low dimensional) subset, which requires nonlinear methods for constructing complex data approximators, and a high-dimensional subset, characterized by measure concentration and simplicity allowing the effective application of linear methods. Manifold learning methods and their generalizations using different mathematical objects (such as graphs) aim at revealing and characterizing the geometry and topology of the low-dimensional part of the datapoint cloud.

Manifold learning methods model multidimensional data as a noisy sample from an underlying generating manifold, usually of a relatively small dimensionality. A classical linear manifold learning method is the principal component analysis (PCA), introduced more than 100 years ago [8]. From the 1990s, multiple generalizations of PCA to non-linear manifolds have been suggested, including injective (when the manifold exists in the same space as the data themselves) methods such as self-organizing maps (SOMs) [9], elastic maps [10,11], regularized principal curves and manifolds [12], and projective (when the projection forms its own space) ones such as ISOMAP [13], local linear embedding (LLE) [14], *t*-distributed stochastic neighbor embedding (t-SNE) [15], UMAP [16], and many others.

The manifold hypothesis can be challenged by the complexity of real-life data, frequently characterized by clusters having complex shapes, branching or converging (i.e., forming loops) non-linear trajectories, regions of varying local dimensionality, high level of noise, etc. In many contexts, the rigorous definition of manifold as underlying model of data structure may be too restrictive, and it might be advantageous to construct data approximators in the form of more general mathematical varieties, for example, by gluing manifolds on their boundaries, thus introducing singularities which can correspond to branching points.

Many methods currently used to learn complex non-linear data approximators are based on an auxiliary object called *k*-nearest neighbor (*kNN*) graph, constructed by connecting each data point to its *k* closest (in a chosen metrics) neighboring points, or similar objects such as ε-proximity graphs. These graphs can be used to re-define the similarity measures between data points (e.g., along the geodesic paths in the graph), and to infer the underlying data geometry.

By contrast, principal graphs, being generalizations of principal curves, are data approximations constructed by injecting graphs into the data space ‘passing through the middle of data’ and possessing some regular properties, such that the complexity of the injection map is constrained [17,18,19]. Construction of principal graphs might not involve computing *kNN* graph-like objects or complete data distance matrix. Principal tree, a term coined by Gorban and Zinovyev in 2007, is the simplest and most tractable type of principal graph [20,21,22,23]. The historical sequence of the main steps of the principal graph methodology development, underlining the contribution and novelty of this paper, is depicted in Table 1.

In this paper, we present a method for constructing principal graphs, called ElPiGraph (Elastic PrincIpal Graphs). As any complex non-linear data approximator, ElPiGraph has to solve two interconnected problems: (a) learning the hidden data geometry and (b) learning the underlying data topology. For the first part, ElPiGraph exploits the pseudo-quadratic elastic energy functional to fit a fixed graph structure to the data. To learn the data topology, ElPiGraph uses a gradient descent-like search in the discrete space of admissible graph structures, defined by the topological graph grammars, i.e., sets of graph rewriting rules. Optimization-based exploration of the graph structure space (as a space of possible data models), equipped with metrics defined by topological grammars, relates ElPiGraph to the information geometry approach broadly understood.

ElPiGraph combines several concepts previously discussed, including elastic energy functional and topological graph grammars [10,20]. However, it improves significantly over existing methodology and implementations, making it capable of effectively modeling real-word applications with datasets containing large numbers of data points. In ElPiGraph, we introduced several improvements related to scalability, robustness to noise (both sampling and background type), ability to construct non tree-like data approximators, and direct control over the complexity of the principal graph. To achieve this result, we introduced the elastic matrix and Laplacian-based penalty (see Methods section) in order to completely vectorize the algorithm’s implementation which is essential for modern programming languages.

ElPiGraph is currently implemented in five programming languages (R, MATLAB, Scala, Java, Python with possibility to use Graphical Processor Units (GPUs)), which makes it easily applicable across scientific domains.

One of the major areas for application of principal graphs is modern molecular biology. For example, recently obtained snapshot distributions of single cells of a developing embryo or an adult organism, when looked in the space of their omics profiles, display complex multidimensional patterns [37,38,39], reflecting continuous changes in the regulatory programs of the cells and their bifurcations during complex cell fate decisions. The existence and biological relevance of such patterns was clearly demonstrated while studying development [40], cellular differentiation [41,42,43], and cancer biology [44]. This stimulated the emergence of a number of tools in the field of bioinformatics for reconstructing so-called “branching cellular trajectories” and “pseudotime” (loosely defined as the length of the geodesic path between two data points along the graph approximating a datapoint cloud) [35,45,46]. Some of these tools exploit the notion of principal curves or graphs explicitly [32,47], while others sometimes use a different terminology closely related to principal graphs or principal trees in its purpose [48,49]. Biology is, however, only one of the possible domains for applicability of principal graphs. They can serve as useful data approximations in other fields of science such as astronomy or political sciences or image processing [11,30].

## 2. Materials and Methods

### 2.1. General Objective of the ElPiGraph as a Data Approximation and Dimensionality Reduction Method

Given a finite set of multi-dimensional vectors *X* = {*X*_i_}, *i* = 1…*N*, in *R^m^* where *N* is the number of data points and *m* is the number of variables, we aim at approximating it by nodes of a graph injected into *R^m^*. The number of the injected graph nodes |*V*| is supposed to be small |*V*|<< *N*, and the configuration of nodes is supposed to be regular and not too complicated in certain sense which depends on the graph topology. The general graph topology is constrained too and in most of the applications is restricted to linear, grid-like, tree-like, or ring-like structures even though more complex graph classes such as cubic complexes can be used [20]. The graph nodes together with the linear segments corresponding to graph edges forms a base space to project data points onto it which provides dimensionality reduction. Geodesic distances defined along the graph edges define new similarity measure for the data points which might significantly differ compared to the distance in the ambient space *R^m^*.

### 2.2. Elastic Graphs: Basic Definitions

Let *G* be a simple undirected graph with a set of nodes *V* and a set of edges *E*. Let |*V|* denote the number of nodes of the graph, and |*E|* denote the number of edges. Let a *k*-*star* in a graph *G* be a subgraph with *k* + 1 nodes *v*_0*,*1*,…,k*_ ∈ *V* and *k* edges {(*v*_0_*, v_i_*)*|i* = 1, …, *k*}. Let *E*^(*i*)^(0), *E*^(*i*)^(1) denote the two nodes of a graph edge *E*^(*i*)^, and *S^(j)^*(0), …, *S^(j)^*(*k*) denote nodes of a *k*-star *S^(j)^* (where *S^(j)^*(0) is the central node, to which all other nodes are connected). Let deg(*v*_i_) denote a function returning the order *k* of the star with the central node *v*_i_ and 1 if *v_i_* is a terminal node. Let φ:*V* → **R***^m^* be a map which is an injection of the graph into a multidimensional space by mapping a node of the graph to a point in the data space. For any *k*-star of the graph *G*, we call its injection ‘harmonic’ if the position of its central node coincides with the mean of the leaf positions, i.e., $\phi \left(central\;node\right)=\frac{1}{k}\sum_{i=1\ldots k}\phi \left(i\right)$, where *i* iterates over leafs. A mapping φ of graph *G* is called harmonic if injections of all *k*-stars of *G* are harmonic.

We define an elastic graph as a graph with a selection of families of *k*-stars *S_m_* and for which all *E*^(*i*)^ ∈ *E* and $S_{m}^{\left(j\right)}$ have associated elasticity moduli *λ_i_ >* 0 and *μ_j_ >* 0. Furthermore, a primitive elastic graph is defined as an elastic graph in which every non-leaf node (i.e., with at least two neighbors) is associated with a *k*-star formed by all the neighbors of the node. All *k*-stars in the primitive elastic graph are in the selection, i.e., the *S_m_* sets are completely determined by the graph structure. Non-primitive elastic graphs are not considered here, but they can be used, for example, for constructing 2D and 3D elastic principal manifolds, where a node in the graph can be a center of two 2-stars, in a rectangular grid [11].

We also define an elastic tree as an acyclic primitive elastic graph.

### 2.3. Elastic Energy Functional and Elastic Matrix

The elastic energy of the graph injection is defined as a sum of squared edge lengths (weighted by the elasticity moduli *λ_i_*) and the sum of squared deviations from harmonicity for each star (weighted by the *μ_j_*)

(1) $$U_{}^{\phi }\left(G\right)=U_{E}^{\phi }\left(G\right)+U_{R}^{\phi }\left(G\right)$$

(2) $$U_{E}^{\phi }\left(G\right)=\underset{E^{\left(i\right)}}{\sum }\left[\lambda_{i}+\alpha \left(\max\left(2,\deg\left(E^{\left(i\right)}\left(0\right)\right),\deg\left(E^{\left(i\right)}\left(1\right)\right)\right)-2\right)\right]{\left(\phi \left(E^{\left(i\right)}\left(0\right)\right)-\phi \left(E^{\left(i\right)}\left(1\right)\right)\right)}^{2}$$

(3) $$U_{R}^{\phi }\left(G\right)=\underset{S_{}^{\left(j\right)}}{\sum }\mu_{j}{\left(\phi \left(S_{}^{\left(j\right)}\left(0\right)\right)-\frac{1}{\deg\left(S_{}^{\left(j\right)}\left(0\right)\right)}\overset{\deg\left(S_{}^{\left(j\right)}\left(0\right)\right)}{\underset{i=1}{\sum }}\phi \left(S_{}^{\left(j\right)}\left(i\right)\right)\right)}^{2}$$

The term describing the deviation from harmonicity in the case of 2-star is a simple surrogate for minimizing the local curvature. In the case of *k*-stars with *k* > 2 it can be considered as a generalization of local curvature defined for a branching point [11].

The α term penalizes the appearance of complex branching points. To illustrate this aspect, let us consider graph structures each having 11 nodes and 10 edges of equal unity length. Then, for example, the 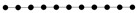 graph is characterized by a $U_{E}^{\phi }\left(G\right)=10\lambda$ contribution to the elastic energy. The graph with one star 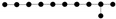 will be characterized by a $U_{E}^{\phi }\left(G\right)=10\lambda +3\alpha$ penalty, 
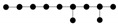
 by $U_{E}^{\phi }\left(G\right)=10\lambda +6\alpha$, and 
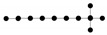
 by $U_{E}^{\phi }\left(G\right)=10\lambda +8\alpha$.

If α = 0 branching is not penalized, while larger values progressively penalize branching, with α ≈ 1 resulting in branching being effectively forbidden. Supplementary Figure S1 illustrates, using the standard iris and a synthetic dataset, how changing this parameter eliminates non-essential branches of a fitted principal tree, up to prohibiting them and simplifying the principal tree structure to a principal curve. Without this penalty term, extensive branches can appear in the regions of data distributions which can be characterized by a ‘thick turn’, i.e., when the increased local curvature of the intrinsic underlying manifold leads to increased local variance of the dataset (Supplementary Figure S1).

**Figure 1:**
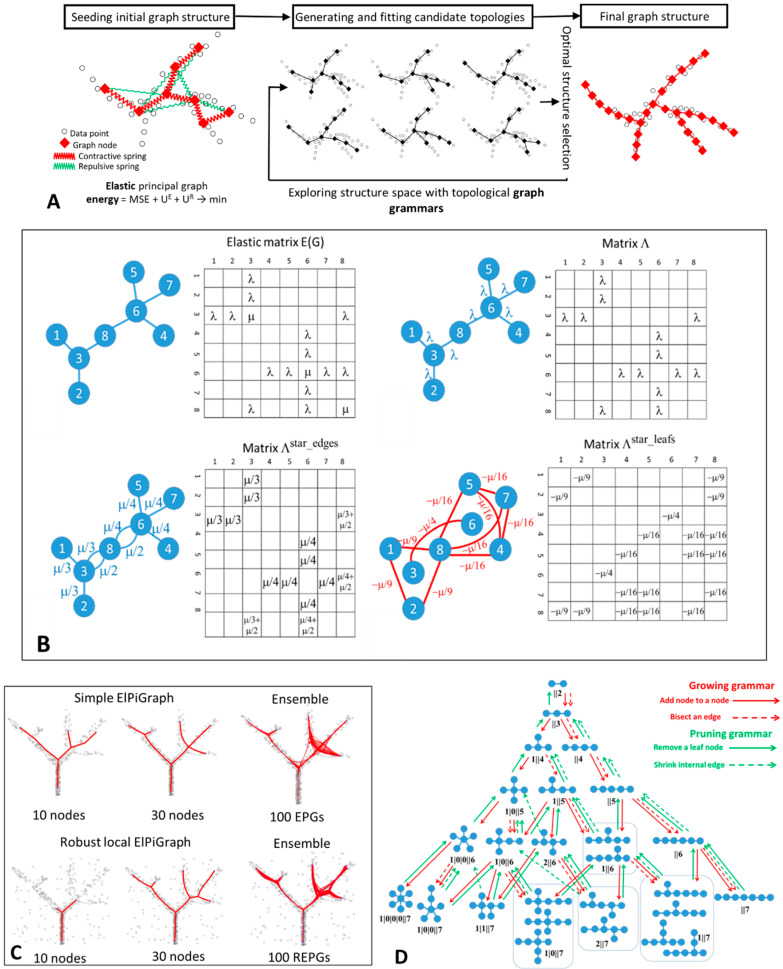
Basic principles and examples of ElPiGraph usage. (**A**) Schematic workflow of the ElPiGraph method. Left, construction of the elastic graph starts by defining the initial graph topology and embedding it into the data space. The graph structure is fit to the data, using minimization of the mean square error regularized by the elastic energy. The elastic energy includes a term reflecting the overall stretching of the graph (symbolically shown as contractive red springs) and a term reflecting the overall bending of the graph branches and the harmonicity of branching points (shown as repulsive green springs). Middle, ElPiGraph explores a large region of the structural space by exhaustively applying a set of graph rewriting rules (graph grammars) and selecting, at each step, the structure leading to the minimum overall energy of the graph embedding. (**B**) Example of elastic matrix for a simple graph and transforming this matrix into several parts used for optimization (see details in the Methods). (**C**) Left and middle, illustration of the robust local workflow of ElPiGraph, which makes it possible to deal with the presence of noise. In the global version, the graph structure is fit to all data points at the same time, while in the local version, the structure is fit to the points in the local graph neighborhood, which expands as the graph grows. Right, an illustration of principal graph ensemble approach: 100 elastic principle graphs are superimposed, each constructed on a fraction of data points randomly sampled at each execution. (**D**) Application of a set of graph grammar rules to generate a set of tree topologies with up to seven nodes.

Note that $U_{R}^{\phi }(G)$ can be re-written as

(4) $$\begin{array}{l} U_{R}^{\phi }\left(G\right)=\underset{S_{}^{\left(j\right)}}{\sum }\frac{\mu_{j}}{\deg\left(S_{}^{\left(j\right)}\left(0\right)\right)}\overset{\deg\left(S_{}^{\left(j\right)}\left(0\right)\right)}{\underset{i=1}{\sum }}{\left(\phi \left(S_{}^{\left(j\right)}\left(0\right)\right)-\phi \left(S_{}^{\left(j\right)}\left(i\right)\right)\right)}^{2}\\ \;\;\;\;\;\;\;-\underset{S_{}^{\left(j\right)}}{\sum }\frac{\mu_{j}}{{\left(\deg\left(S_{}^{\left(j\right)}\left(0\right)\right)\right)}^{2}}\overset{\deg\left(S_{}^{\left(j\right)}\left(0\right)\right)}{\underset{i=1,p=1,i<>p}{\sum }}{\left(\phi \left(S_{}^{\left(j\right)}\left(i\right)\right)-\phi \left(S_{}^{\left(j\right)}\left(p\right)\right)\right)}^{2} \end{array}$$

i.e., the term $U_{R}^{\phi }(G)$ can be considered as a sum of the energy of elastic springs connecting the star centers with its neighbors (with elasticity moduli *μ_j_*/*deg*(*S*^(*j*)^)) and the energy of negative (repulsive) springs connecting all non-central nodes in a star pair-wise (with negative elasticity moduli*-μ_j_*/(*deg*(*S*^(*j*)^))^2^). The resulting system of springs, whose energy is minimized, is shown in Figure 1A,B.

The elasticity of the principal graph contains three parts: positive springs corresponding to elasticity of graph edges, negative repulsive springs describing the node repulsion, positive springs representing the correction term such that the smoothing would correspond to the deviation of φ from a harmonic map (Figure 1B).

For vectorized implementations of the algorithm it is convenient to describe the structure and the elastic properties of the graph by elastic matrix *EM*(*G*). The elastic matrix is a |*V|×*|*V|* symmetric matrix with non-negative elements containing the edge elasticity moduli *λ_i_* at the intersection of rows and lines, corresponding to each pair *E*^(*i*)^(0), *E*^(*i*)^(1), and the star elasticity module *μ_j_* in the diagonal element corresponding to *S*^(*j*)^ (0). Therefore, *EM*(*G*) can be represented as a sum of two matrices Λ and *M*.

(5) EM(G)=Λ(G)+M(G)

where Λ is an analog of the weighted adjacency matrix for the graph *G*, with elasticity moduli playing the role of weights, and *M*(*G*) is a diagonal matrix having non-zero values only for nodes that are centers of starts, in which case the value indicates the elasticity modulus of the star. An example of elastic matrix is shown in Figure 1B.

It is also convenient to transform *M*(*G*) into two auxiliary matrices $\Lambda_{}^{star\_edges}$ and $\Lambda_{}^{star\_leafs}$. $\Lambda_{}^{star\_edges}$ is a weighted adjacency matrix for the edges connected to star centers, with elasticity moduli *μ_j_/k_j_,* where *k_j_* is the number of edges connected to the *j*th star center. $\Lambda_{}^{star\_leafs}$ is a weighted adjacency matrix for the negative springs (shown in green in Figure 1A and in red in Figure 1B), with elasticity moduli −*μ_j_/(k_j_*)^2^. An example of transforming the elastic matrix *EM*(*G*) into three weighted adjacency matrices Λ, $\Lambda_{}^{star\_edges}$, $\Lambda_{}^{star\_leafs}$ is shown in Figure 1B.

For the system of springs shown in Figure 1A, if one applies a distributed force to the nodes, then the node displacement will be described by a matrix which is a sum of three Laplacians.

(6) $$\tilde{L}\left(EM\left(G\right)\right)=L\left(\Lambda \right)+L\left(\Lambda_{}^{star\_edges}\right)+L\left(\Lambda_{}^{star\_leafs}\right).$$

We remind that a Laplacian matrix for a weighted adjacency matrix *A* is computed as $L{\left(A\right)}_{ij}=\delta_{ij}\overset{}{\underset{k}{\sum }}A_{kj}-A_{ij}$, where $\delta_{ij}$ is the Kronecker delta. Matrix (6) is positive semi-definite by construction because L(Λ) is positive semi-definite and the sum $L\left(\Lambda_{}^{star\_edges}\right)+L\left(\Lambda_{}^{star\_leafs}\right)$ is positive semi-definite because (3) is by construction a positive semi-definite quadratic form.

### 2.4. Elastic Principal Graph Optimization

Assume that we have defined a partitioning *K* of all data points such that *K*(*i*) = $\arg\min_{j=1\ldots \left|V\right|}\|X_{i}-\phi (V_{j})\|$ is an index of a node in the graph which is the closest to the *i*^th^ data point among all graph nodes. We define the optimization functional for fitting a graph to data as a sum of the truncated approximation error and the elastic energy of the graph

(7) $$U_{}^{\phi }\left(X,G\right)=\frac{1}{\sum w_{i}}\overset{\left|V\right|}{\underset{j=1}{\sum }}\underset{K\left(i\right)=j}{\sum }w_{i}\cdot \min\left(\|X_{i}-\phi {\left(V_{j})\right\|}^{2},R_{0}^{2}\right)+U_{}^{\phi }\left(G\right),$$

where *w_i_* is a weight of the data point *i* (can be equal one for all points), |V| is the number of nodes, ||..|| is the usual Euclidean norm.

$R_{0}$ is a trimming radius that can be used to limit the effect of points distant from the current configuration of the graph [31]. Data points located farther than $R_{0}$ from any graph node position do not contribute, for a given data point partitioning, to the optimization equation in Algorithm 1. However, these data points might appear at a distance smaller than $R_{0}$ at the next algorithm iteration: therefore, it is not equivalent to permanently pre-filtering ‘outliers’. The approach is similar to the ‘data-driven’ trimmed *k*-means clustering [50]. Alternative ways of constructing robust principal graphs include using piece-wise quadratic subquadratic error functions (PQSQ potentials) [51], which uses computationally efficient non-quadratic error functions for approximating a dataset. These two approaches will be implemented in the future versions of ElPiGraph.

The objective of the basic optimization algorithm is to find a map φ:*V→* **R***^m^* such that *U^φ^*(*X*,*G*) → min over all possible elastic graph *G* injections into **R***^m^*. In practice, we are looking for a local minimum of *U*^φ^(*X*,*G*) by applying the standard splitting-type algorithm, which is described by the pseudo-code provided below. The essence of the algorithm (similar to the simplest *k*-means clustering) is to alternate between 1) computing the partitioning *K* given the current estimation of the map φ and 2) computing new map φ provided the partitioning *K*. The first task is a standard neighbor search problem, while the second task is solving a system of linear equations of size |*V*| (see Algorithm 1).
**Algorithm 1** Base graph optimization for a fixed structure of the elastic graph(1)Initialize the graph G, its elastic matrix EM(G) and the map φ(2)Compute the matrix $\tilde{L}\left(EM\left(G\right)\right)$
(3)Partition the data by proximity to the embedded nodes of the graph (i.e., compute the mapping K:{X} →{V} of a data point i to a graph node j)(4)Solve the following system of linear equations to determine the new map φ$$\sum_{j=1}^{\left|V\right|}\left(\frac{\sum_{\left\{K\left(i\right)=j\right\}}w_{i}}{\sum_{i=1}^{\left|V\right|}w_{i}}\delta_{ij}+\tilde{L}{\left(EM\left(G\right)\right)}_{ij}\right)\phi \left(V_{j}\right)=\frac{1}{\sum_{i=1}^{\left|V\right|}w_{i}}\sum_{K\left(i\right)=j}^{}w_{i}X_{i},$$ where $\delta_{ij}$ is the Kronecker delta.Iterate 3–4 till the map φ does not change more than ε in some appropriate measure of difference between two consecutive iterations.

### 2.5. Graph Grammar-Based Approach for Determining the Optimal Graph Topology

A graph-based data approximator should deal simultaneously with two inter-related aspects: determining the optimal topological structure of the graph and determining the optimal map for embedding this graph topology into the multidimensional space. An exhaustive approach would be to consider all the possible graph topologies (or topologies of a certain class, e.g., all possible trees), find the best mapping of all them into the data space, and select the best one. In practice, due to combinatorial explosion, testing all the possible graph topologies is feasible only when a small number of nodes is present in the graph, or under restrictive constraints (e.g., only trivial linear graphs, or assuming a restricted set of topologies with a pre-defined number and types of branching). Determining a globally optimal injection of a graph with a given topology is usually challenging, because of the energy landscape complexity. This means that, in practice, one has to use an optimization approach in which both graph topology and the injection map should be learned simultaneously.

A graph grammar-based approach for constructing such an algorithm was suggested [17]. The algorithm starts from an initial graph *G_0_* and an initial map φ_0_(*G*_0_). In the simplest case, the initial graph can be initialized by two nodes and one edge and the map can correspond to a segment of the first principal component.

Then a set of predefined grammar operations which can transform both the graph topology and the map, is applied starting from a given pair {*G_i_*, φ*_i_*(*G_i_*)}. Each grammar operation Ψ*^p^* produces a set of new graphs and their injection maps possibly taking into account the dataset *X*

$$\{\{D_{}^{k},\phi (D_{}^{k})\},k=1\ldots s\}=\Psi_{}^{p}(\left\{G_{i}^{},\phi_{i}^{}(G_{i}^{})\right\},X).$$

Given a pair {*G_i_*, φ*_i_*(*G_i_*)}, a set of *r* different graph operations {Ψ^1^*,…,* Ψ*^r^*} (which we call a ‘graph grammar’), and an energy function ${\bar{U}}_{}^{\phi }\left(X,G\right)$, at each step of the algorithm the energetically optimal candidate graph embedment is selected as

$$\left\{G_{i+1},\phi_{i+1}\left(G_{i+1}\right)\right\}={\mathrm{argmin}}_{\left\{D^{k},\;\phi \left(D^{k}\right)\right\}}\left\{{\bar{U}}^{\phi \left(D^{k}\right)}\left(D^{k},X\right):\left\{D^{k},\;\phi \left(D^{k}\right)\right\}\in \underset{p=1\ldots r}{\cup }\Psi^{p}\left(\left\{G_{i},\phi_{i}\left(G_{i}\right)\right\},X\right)\right\}$$

where $\left\{D_{}^{k},\phi \left(D_{}^{k}\right)\right\}$ is supposed to be optimized (fit to data) after the application of a graph grammar using Algorithm 1 with initialization suggested by the graph grammar application (see below).

The pseudocode for this algorithm is provided below:
**Algorithm 2** graph grammar based optimization of graph structure and embedment(1)Initialize the current graph embedment by some graph topology and some initial map {*G_0_*, φ*_0_*(*G*_0_)}.(2)For the current graph embedment {*G_i_*, φ*_i_*(*G_i_*)}, apply all grammar operations from a grammar set {Ψ^1^,…, Ψ^r^}, and generate a set of s candidate graph injections $\{D_{}^{k},\phi \left(D_{}^{k}\right),\;k=1\ldots s\}.$
(3)Further optimize each candidate graph embedment using Algorithm 1, and obtain a set of s energy values $\left\{{\bar{U}}^{\phi \left(D_{}^{k}\right)}\left(D_{}^{k}\right)\right\}$
(4)Among all candidate graph injections, select an injection map with the minimum energy as $\{G_{i+1}^{},\phi_{i+1}^{}(G_{i+1}^{})\}.$
Repeat 2–4 until the graph contains a required number of nodes.

Note that the energy function ${\bar{U}}_{}^{\phi }\left(X,G\right)$ used to select the optimal graph structure is not necessarily the same energy as (1–3) and can include various penalties to give less priority to certain graph configurations (such as those having excessive branching). Separating the energy functions $U_{}^{\phi }\left(X,G\right)$, used for fitting a fixed graph structure to the data, and ${\bar{U}}_{}^{\phi }\left(X,G\right)$, used to select the most favorable graph configuration, allows achieving better flexibility in defining the strategy for selecting the most optimal graph topologies.

One of the simplest and practical case of topological graph grammar is applying a combination of two grammar operations ‘bisect an edge’ and ‘add a node to a node’ as defined below.
GRAPH GRAMMAR OPERATION ‘BISECT AN EDGE’GRAPH GRAMMAR OPERATION ‘ADD NODE TO A NODE’  Applicable to: any edge of the graph  Applicable to: any node of the graph   Update of the graph structure: for a given edge {A,B}, connecting nodes A and B, remove {A,B} from the graph, add a new node C, and introduce two new edges {A,C} and {C,B}.  Update of the graph structure: for a given node A, add a new node C, and introduce a new edge {A,C}   Update of the elasticity matrix: the elasticity of edges {A,C} and {C,B} equals elasticity of {A,B}.Update of the elasticity matrix:  *if A is a leaf node (not a star center) then*  the elasticity of the edge {A,C} equals to the edge connecting A and its neighbor, the elasticity of the new star with the center in A equals to the elasticity of the star centered in the neighbor of A. If the graph contains only one edge then a predefined values is assigned.  *else*  the elasticity of the edge {A,C} is the mean elasticity of all edges in the star with the center in A, the elasticity of a star with the center in A does not change.   Update of the graph injection map: C is placed in the mean position between the positions of A and B.   Update of the graph injection map:  *if A is a leaf node (not a star center) then*  C is placed at the same distance and the same direction as the edge connecting A and its neighbor,  *else*  C is placed in the mean point of all data points for which A is the closest node

The application of Algorithm 2 with a graph grammar containing only the ‘bisect an edge’ operation, and a graph composed by two nodes connected by a single edge as initial condition, produces an elastic principal curve.

The application of Algorithm 2 with a graph grammar containing only the ‘bisect an edge’ operation, and a graph composed by four nodes connected by four edges without branching, produces a closed elastic principal curve (called elastic principal circle, for simplicity).

The application of Algorithm 2 with a growing graph grammar containing both the ‘bisect an edge’ and the ‘add a node to a node’ operations and a graph composed by two nodes connected by a single edge as initial condition produces an elastic principal tree.

In the case of a tree or other complex graphs, it is advantageous to improve the Algorithm 2 by providing an opportunity to ‘roll back’ the changes of the graph structure. This gives an opportunity to get rid of unnecessary branching or to merge split branches created in the history of graph optimization, if this is energetically justified (see Figure 1D). This possibility can be achieved by introducing a shrinking grammar. In the case of trees, the shrinking grammar consists of two operations ‘remove a leaf node’ and ‘shrink internal edge’ (defined below). Then the graph growth can be achieved by alternating two steps of application of the growing grammar with one step of application of the shrinking grammar. In each such cycles, one node will be added to the graph.
GRAPH GRAMMAR OPERATION ‘REMOVE A LEAF NODE’GRAPH GRAMMAR OPERATION ‘SHRINK INTERNAL EDGE’  Applicable to: node A of the graph with deg(A) = 1  Applicable to: any edge {A,B} such that deg(A) > 1 and deg(B) > 1.  Update of the graph structure: for a given edge {A,B}, connecting nodes A and B, remove edge {A,B} and node A from the graph   Update of the graph structure: for a given edge {A,B}, connecting nodes A and B, remove {A,B}, reattach all edges connecting A with its neighbors to B, remove A from the graph.   Update of the elasticity matrix:  *if B is the center of a 2-star then*  put zero for the elasticity of the star for B (B becomes a leaf)  *else*  do not change the elasticity of the star for B  Remove the row and column corresponding to the vertex A  Update of the elasticity matrix:  The elasticity of the new star with the center in B becomes the average elasticity of the previously existing stars with the centers in A and B  Remove the row and column corresponding to the vertex A   Update of the graph injection map: all nodes besides A keep their positions.   Update of the graph injection map: B is placed in the mean position between A and B. 

### 2.6. Computational Complexity of ElPiGraph

The computational complexity of ElPiGraph algorithm can be evaluated as follows. Fitting each fixed graph structure with *s = |V|* number of nodes to a set of *N* data points in *m* dimensions is of complexity O(*Nsm* + *ms*^2^). The first term O(*Nsm*) comes from the simple nearest neighbor search where each data point is attributed to the closest graph node position. The second term O(*ms*^2^) comest from the solution of the system of *s* linear equations with sparse matrix *m* times. With usual situation *N* >> *s,* the first term always dominates the complexity.

At each step of application of graph grammars, a set of candidate graph structures is generated, the number of which depends on the nature of the graph grammar. We can distinguish local and non-local graph grammars. The local grammars apply a graph re-writing rule only to one element of the graph (node or edge) or to a compact neighborhood of an element (e.g., a node and all its neighboring nodes). In this case the number of candidate graph structures grows linearly with the number of elements, which is approximately proportional to *s*. The non-local grammars can deal with distant combinations of graph elements (e.g., it can be applied to all pairs of graph nodes). In this case, the number of candidate graph structures can grow polynomially with *s*. Since the current implementation of ElPiGraph considers only local grammar operations, we assume that the number of candidate graph structures depends linearly on *s*.

Therefore, the total complexity of ElPiGraph algorithm which grows a principal graph from a small number of nodes can be estimated from summation of the series $\sum_{i=1\ldots s}s\times O\left(Nsm\right)=O\left(Nms^{3}\right)$. It remains linear with the number of points *N* and dimensionality *m* but is polynomial with the number of nodes *s*, which is caused by testing a large number of candidate graph structures in the process of gradient descent-like search for the optimal topology.

In practical applications, when there is a need to construct large principal graphs for big datasets, the polynomial complexity of ElPiGraph can be prohibitive. In this case, there exist multiple options (besides application of GPUs) to speed up the algorithm, some of which are listed below:

(1) Effective reduction of the number of points in the dataset, e.g., by applying pre-clustering. The new datapoints represent the centroids of clusters weighted accordingly to the number of points in each cluster. Alternatively, ElPiGraph can use stochastic approximation approach, by exploiting a subsample of data at each step of growth.
(2) Application of accelerated strategies for the nearest neighbor search step (with the number of neighbors = 1) between graph node positions and data points. In relatively small dimensions and large graphs, the standard use of kdtree method can be already beneficial. The new partitioning can exploit the results of the previous partitioning in order to prevent recomputing all distances, similar to the fast versions of *k*-means algorithm [52,53]. Various strategies of approximate nearest neighbor search can be also exploited.
(3) Reducing the number of tested candidate graph topologies using simple heuristics. For example, one can test at each application of ‘Bisect an edge’ grammar operation only *k* longest edges, which most probably will be selected as optimal, with small and fixed *k*. Similarly, ‘Add a node’ grammar operations can be applied to the several most charged with data points nodes.

Some of these approaches have been tested on synthetic data and the resulting speed up is reported further in the text.

### 2.7. Strategies for Graph Initialization

The construction of elastic principal graphs in ElPiGraph can be organized either by graph growth (similar to divisive clustering) or by shrinking the graph (similar to agglomerative clustering) or by exploring possible graph structures having the same number of nodes. These different strategies can be achieved by specifying the appropriate graph grammars set. The initial graph structure can have a strong influence on the final graph mapping to the data space and its topology because of the presence of multiple local minima in the optimization criteria. Hence it is very important to define problem-specific strategies that are helpful to guess the initial graph structure. Under most circumstances the graph topology exploration procedure of ElPiGraph is capable of improving the initial graph structure despite the fact that this guess can be too complex, or too coarse-grained.

The default setting of ElPiGraph initializes the graph with the simplest configuration containing two nodes oriented along the first principal component: this initialization is able to correctly fit data topology in relatively simple cases. Other initializations are advised in more complex cases: for example, applying pre-clustering and computing (once) the minimal spanning tree between cluster centroids can be used for the initialization of the principal tree. This approach is used by the STREAM pipeline [33], while in MERLoT pipeline, the principal tree is initiated by finding a so called ‘scaffold tree’ from a low-dimensional distance matrix with further application of ElPiGraph [34].

When using a finite trimming radius *R_0_* value, the advised strategy is graph growing which can be initialized by 1) a rough estimation of local data density in a limited number of data points and 2) placing two nodes, one into the data point characterized by the highest local density and another one placed into the data point closest to the first one (but not coinciding).

In case of existence of several well-separable clusters in the data, with the distance between them larger than $R_{0}$, the robust version of ElPiGraph can approximate the principal graph only for one of them, completely disregarding the rest of the data. In this case, the approximated part of the data can be removed and the robust ElPiGraph can be re-applied. For example, this procedure will construct a second (third, fourth, etc.) principal tree. Such an approach will approximate the data by disconnected ‘principal forest’.

### 2.8. Fine-Tuning the Final Graph Structure

After the core ElPiGraph procedure produces a tree, it can be desirable to fine-tune it in order to better answer requirements of certain applications. These fine-tuning operations are usually implemented as graph-rewriting rules: however, they are applied once without further graph optimizations. Below we give two examples of fine-tuning operations. For other examples, one can refer to the ElPiGraph documentations or supplementary illustration in [33].

The ElPiGraph algorithm is designed to place nodes at the centre of set of points. As a consequence of this, the leaf nodes of a principal graph might not capture the extreme points of the data distribution. This is not ideal when the branch of a principal graph is used to rank data points accordingly to the projection onto it. Hence, ElPiGraph includes a function that can be used to extend the leaf nodes by extrapolation.

Under certain circumstances, it might be necessary to simplify a principal graph by removing edges or nodes that capture only a small subset of points. For this purpose, ElPiGraph includes a function that removes edges on which only a limited number of points are projected. The nodes of the graph are then removed and locally re-optimized when necessary.

### 2.9. Choice of the Core ElPiGraph Parameters

The core ElPiGraph algorithm uses four parameters with well-defined effect on its behavior:

λ—coefficient of ‘stretching’ elasticity;

μ—coefficient of ‘bending’ elasticity;

α—parameter controlling graph complexity;

*R*_0_—trimming radius for MSE-based data approximation term.

Base parameters of elasticity control the general regular properties of the graph injection map. λ effectively constraints the total length of edges in the graph as well as penalizes the deviation from the uniform edge length distribution. μ penalizes deviations of graph *k*-stars from harmonicity. For linear branches this means deviation from linearity, therefore large values of μ lead to graphs having piece-wise linear injection maps with close to harmonic *k*-stars. The values of λ and μ are dimensionless and normally do not depend on scaling the dataset variables. Empirically, it is shown that μ should be made at least one order of magnitude larger than λ. Typical starting values for λ are 10^−2^, however, the exact value can depend on the requirements for the resulting graph properties.

Graph complexity control parameter α allows avoiding appearance of *k*-stars in the graph with large *k*s, a usual problem when the principal graph is constructed in large dimensions. In case of excessive branching, it is recommended to set up the branching control parameter α to a small value (e.g., 0.01). Changing the value of α from 0 to a large value (e.g., 1) allows gradual change from the absence of excessive branching penalty to the effective interdiction of branching (thus, constructing a principal curve instead of a tree as a result).

**Figure 2:**
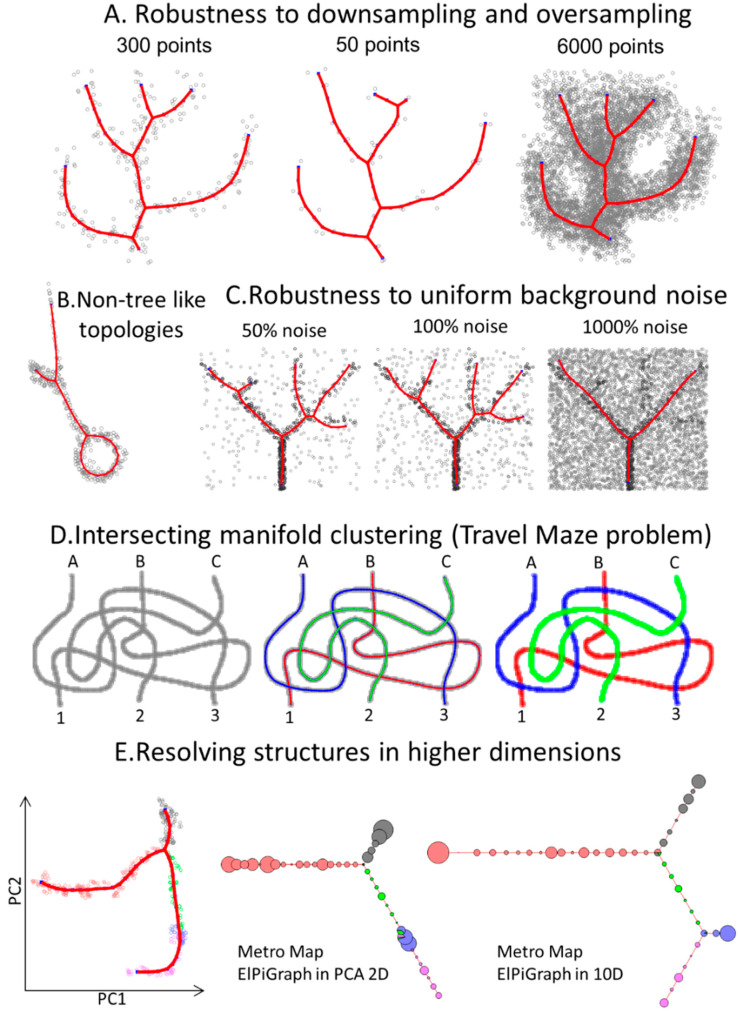
Synthetic examples showing features of ElPiGraph. (**A**) ElPiGraph is robust with respect to downsampling and oversampling of a dataset. Here, a reference branching dataset [32] is downsampled to 50 points (middle) or oversampled by sampling 20 points randomly placed around each point of the original dataset. More systematic study of the down/over-sampling effect on robustness of the principal graph is shown in Supplementary Figure S2. (**B**) ElPiGraph is able to capture non-tree like topologies. Here, the standard set of principal tree graph grammars was applied to a graph initialized by four nodes connected to a ring. (**C**) ElPiGraph is robust to large amounts of uniformly distributed background noise. Here, the initial dataset from Figure 1 is shown as black points, and the uniformly distributed noisy points are shown as grey points. ElPiGraph is blindly applied to the union of black and grey points. (**D**) ElPiGraph is able to solve the problem of learning intersecting manifolds. On the left, a toy dataset of three uniformly sampled curves intersecting in many points is shown. ElPiGraph starts by learning a principal curve using the local version several times, each time on a complete dataset. However, for each iteration, ElPiGraph is initialized by a pair of neighboring points not belonging to points already captured by a principal curve. The fitted curves are shown in the middle of the point distribution by using different colors, and the clustering of the dataset based on curve approximation is shown on the right. (**E**) Approximating a synthetic ten-dimensional dataset with known branch structure (with different colors indicating different branches), where one of the branches (blue one) extrude into higher dimensions and collapses with other branches when projected in 2D by principal component analysis (left). Middle, being applied in the space of two first principal components, ElPiGraph does not recover the branch, while it is captured when the ElPiGraph is applied in the complete 10-dimensional dataset (right). In both cases, the principal tree is visualized using metro map layout [21], and a pie chart associated to each node of the graph indicates the percentage of points belonging to the different populations. The size of the pie chart indicates the number of points associated with each node.

Using less than infinite trimming radius *R_0_* value changes the way the principal graph is grown. In this case it explores the dataset starting from a small fragment of it by ‘crawling’ to the neighbor data points simultaneously fitting the local features of data topology. With properly chosen $R_{0}$, the ElPiGraph algorithm can tolerate significant amount of uniformly distributed background noise (see Figure 2C) and even deal with self-intersecting data distributions (Figure 2D). ElPiGraph includes a function for estimating the coarse-grained radius of the data based on local analysis of density distribution, which can be used as an initial guess for the *R*_0_ value. An alternative initial guess for the trimming radius can be obtained by taking the median of the pairwise data point distances distribution.

### 2.10. Principal Graph Eensembles and Consensus Principal Graph

Resampling techniques are often used to determine the significance of analyses applied to complex datasets [54]. ElPiGraph can be also used in conjunction with resampling (i.e., random sampling of *p*% of data points and applying ElPiGraph *k* times) to explore the robustness of the constructed principal graph. Resampling produces a principal graph ensemble, i.e., a set of *k* principal graphs. Figure 1C shows a simple example of using data resampling in order to construct multiple principal trees (*k* = 100 and *p* = 90%). From this example, it is possible to judge how a different level of uncertainty is associated with different parts of the tree and to explore the uncertainty associated with branching points.

Besides resampling, other approaches can be applied in order to build the principal graph ensemble. For example, it can be built from multiple initializations of the graph structure, or by varying the parameters of ElPiGraph in certain range as it is illustrated in Section 3.4.

In complex examples, a principal graph ensemble can be used to infer a consensus principal graph, i.e., a principal graph obtained by recapitulating the information from the graph ensemble, and to discover in such a way emergent complex topological features of the data. For example, a consensus principal graph constructed by combining an ensemble of trees may contain loops, which are not present in any of the constructed principal trees (Figure 5).

ElPiGraph package contains a set of functions to assemble consensus principal graphs which uses simple graph abstraction approach. Briefly, the currently implemented approach consists in (1) generating a certain number of elastic principal graphs using resampling strategy; (2) matching the nodes from multiple principal graphs and combining them into node clusters such that each cluster would contain certain minimal number of nodes; (3) connecting the node clusters using the edges of individual principal graphs, such that each edge in the consensus principal graph aggregates a certain minimal number of individual principal graph edges; (4) removing isolated nodes from the consensus graph; (5) delete too short or too long edges from the consensus graph preserving the graph connectivity. The final structure of the consensus graph can be further optimized using the ElPiGraph Algorithm 1. We envisage further improvement of the consensus principal graph construction methodology, exploiting methods of graph alignment and introducing appropriate metric structure in the graph ensemble, in order to exclude atypical graph topologies.

### 2.11. Code Availability

The ElPiGraph method is implemented in several programming languages:

- R from https://github.com/sysbio-curie/ElPiGraph.R
- MATLAB from https://github.com/sysbio-curie/ElPiGraph.M
- Python from https://github.com/sysbio-curie/ElPiGraph.P. Note that Python implementation provides support of GPU use.

A Java implementation of ElPiGraph is available as part of VDAOEngine library (https://github.com/auranic/VDAOEngine/) for high-dimensional data analysis developed by AZ. The Java implementation of ElPiGraph is not actively developed and the implementation of ElPiGraph in Java does not scale as good as other implementations.

A Scala implementation is available from https://github.com/mraad/elastic-graph.

All the implementations contain the core algorithm as described in this paper. However, the different implementations differ in the set of functionalities improving the core algorithm such as robust local version of the algorithm, explicit complexity control, multicore, or GPU support.

In the future, ElPiGraph implementation in Python will serve as the reference one, and a regular effort will be made to synchronize the ElPiGraph R version with the reference implementation.

The code used to perform the analysis, to generate the figures, and interactive versions of some of the figures are available from: https://sysbio-curie.github.io/elpigraph/.

## 3. Results

### 3.1. Approximating Complex Data Topologies in Synthetic Examples

To showcase the flexibility, robustness, and scalability of ElPiGraph, we applied it to different use cases. First, we considered a synthetic 2D dataset describing a circle connected to a branching structure. ElPiGraph was able to recover the target structure when the appropriate grammar rules are specified (Figure 2B). The possibility to use various grammar combinations and initial approximations gives ElPiGraph flexibility in approximating datasets with complex topologies. In the simplest case, it requires the a priori defined topology type (e.g., curve, circular, tree-like) of the dataset to approximate. As discussed later, ElPiGraph is able to construct an appropriate data approximator when no information is available on the underlying topology of the data by using the consensus principal graph approach. Use of non-local graph grammars can in principle make learning complex data topologies more efficient, which will be the subject of our future work. It should be noted that such approaches must be accompanied by efficient ways to penalize excessive complexity of principal graphs as discussed in [17]: otherwise, the complexity of the data approximator might easily become comparable with the complexity of the data point cloud itself.

We explored the robustness of ElPiGraph to down-and oversampling. For a simple benchmark example of a branching data distribution in 2D (Figure 2A), we selected a fraction of data points (15%) or generated 20× more points around each of the existing ones in the original dataset. Our algorithm is able to properly recover the structure of the data, regardless of the condition being considered, with only minimal differences in the position of the branching points due to the loss of information associated with downsampling. Nevertheless, small subsamples can, in some cases, lead to large changes in the graph topology, especially when the number of graph nodes becomes comparable with the number of data points (Supplementary Figure S2).

Then, we tested the robustness of ElPiGraph to the presence of uniform background noise covering the branching data pattern (Figure 2C). As the percentage of noisy points increases, certain features of the data are not captured by the approximator, as expected. However, even in the presence of a striking amount of noise, ElPiGraph is capable of partly recovering the underlying data distribution. Note that, in the examples of Figure 2C, the tree is being constructed starting from the densest part of the data point cloud.

Disentangling intersecting curvilinear manifolds is a hard problem (sometimes called the ‘Travel Maze problem’) that has been described in different fields, particularly in computer vision [55]. The intrinsic rigidity of elastic graph in combination with a trimmed approximation error enables ElPiGraph to solve the Travel Maze problem quite naturally. This is possible because the local version of the elastic graph algorithm is characterized by a certain persistency in choosing the direction of the graph growth. Indeed, when presented with a dataset corresponding to a group of three threads intersecting on the same plane, ElPiGraph is able to recognize them and cluster the data points accordingly, hence associating different paths to the different threads (Figure 2D).

As demonstrated by the widespread use of tools like PCA, tSNE, LLE, or diffusion maps, dimensionality reduction can be a powerful tool to better visualize the structure of data. However, some data features can be lost as the dimension of the dataset is being reduced. This can be particularly problematic if dimensionality reduction is used as an intermediate step in a long analytical pipeline. To illustrate this phenomenon, we generated a 10-dimensional dataset describing an a priori known branching process. When the points are projected into a 2D plane induced by the first two principal components, one of the branches collapses and becomes indistinguishable (Figure 2E). This effect is due to the branch under consideration being essentially orthogonal to the plane formed by the first two principal components. As expected, ElPiGraph is unable to capture the collapsed branch, when it is used on the 2D PCA projection of the data. However, the branch is correctly recovered when ElPiGraph is run using all 10 dimensions. In this particular case, applying non-linear dimensionality reduction (e.g., tSNE) could make the ‘hidden’ branch more visible in 2D at the cost of distorting the underlying data geometry. Nevertheless, this situation can be reproduced with any data dimensionality technique (for examples, see [56]).

### 3.2. Benchmarking ElPiGraph

**Figure 3:**
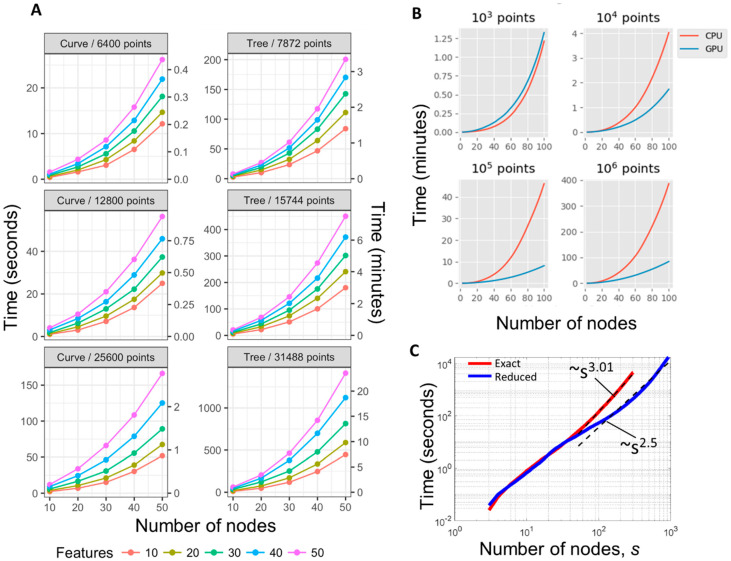
Computational performance of ElPiGraph. (**A**) Time required to build principal curves and trees (*y*-axis) with a different number of nodes (*x*-axis) using the default parameters across synthetic datasets containing different numbers of points having a different number of dimensions (color scale), using single CPU. (**B**) Demonstrating computational advantages of using a combination of GPU and CPU in ElPiGraph computations. Starting from 10k data points, using GPU becomes advantageous. The computational experiment was done using Google Colab environment. (**C**) Empirical performance scaling of ElPiGraph on a 12k data points in 5D example. The exact ElPiGraph algorithm scales as polynome of degree 3, while the approximate reduced algorithm with a limitation on the number of tested candidate structures scales as *s*^2.5^ in the *s* = 50…1000 range, where s is the number of nodes in the principal graph.

The core functionality of ElPiGraph is based on a fast optimization Algorithm 1 which allows the method to scale well to large datasets. ElPiGraph can also take advantage of multiprocessing to further speed up the computation if necessary. When run on a single core, ElPiGraph is able to reconstruct principal curves in few minutes for tens of thousands points in tens of dimensions (Figure 3A). The new Python implementation of ElPiGraph is able to use a combination of CPU and GPU which gives significant benefit in computational performance starting from 10,000 data points in relatively high dimension: we can observe several-fold acceleration for a large dataset compared to the previously published *R* version using only CPU, see Figure 3B. Running ElPiGraph to construct large graphs with several hundreds of nodes showed polynomial dependence of the computational time vs. the number of nodes with fitted polynome of degree 3.01 which is very close to the theoretical estimate (see Figure 3C).

We tested the computational performance of ElPiGraph in the case of using approximate heuristic modifications of the initial ElPiGraph algorithm. For example, we implemented an option to test a fixed number of graph candidates *k* at each step of generating the graph structure candidates. For the operation ‘bisect an edge’ we selected *k* longest edges, and for the operation ‘add a node to a node’ we selected *k* nodes with the largest number of data points associated, as in both cases we expect their local modifications to have the largest impact on the value of the elastic energy. Using such an approximation, ElPiGraph can fit a large number of nodes in reasonable times (see Figure 3C, for *k* = 20, where ElPiGraph in 4 hours builds a principal graph with 1000 nodes on example with 12k data points in 5D, using only CPU). We also systematically checked if the principal graphs produced with this heuristic are similar to the ones produced with the exact algorithm, and this was the case. In particular, we tested moderate size 100 branching data distribution examples (constructed as described below), and found that the values of the elastic energy are correlated at *r* = 0.98, and in 60% of cases the number of detected stars was the same between the exact and reduced methods, while in the rest 40% the difference was in only one detected star. There was a weak trend for the reduced ElPiGraph version to produce more stars, which in most cases could be matched to the ground truth star positions. The last feature can be even advantageous in certain analyses. Accordingly to our theoretical complexity estimate, this heuristic scales as $O\left(Nms^{2}\right).$ Empirically, however, the scaling of the heuristic was superquadratic which was probably caused by some overheads related to dealing with large graphs (e.g., their slower convergence or superquadratic cost of solving the large systems of linear equations).

Computational performance is only one of the features of any algorithm fitting graphs to data. Of course, the main criterion for comparison is the quality of the reconstructed graph topology with respect to a hypothetical ‘true’ data structure (otherwise any simple heuristic can beat a sophisticated algorithm producing reliable results). Such benchmarking is inevitably specific to the nature of the data and the type of distributions; therefore, base algorithms should be tested together with domain-specific data pre-processing steps. For single cell genomic data several comprehensive efforts have been made in order to compare existing approaches, based on synthetic or real data. As a non-specialized machine learning method, ElPiGraph performed well in the recent large-scale benchmarking of cell trajectory inference methods in three categories (linear, circular, tree-like topology), using synthetic datasets with known ground-truth underlying data topology [35]. However, in these tests ElPiGraph was used with the initialization from the simplest graph, without trying to guess the structure adapted to the data. As a part of STREAM pipeline, which provided more advanced initial graph structure guess, ElPiGraph was competitive compared to 10 recently suggested approaches when applied to several real-life datasets [33].

Here, we developed an approach to compare ElPiGraph, as a general-purpose machine learning method, with its closest principal graph approach, without taking into account the specifics of single cell data. Thus, we compared ElPiGraph with SimplePPT [22] and DDRTree [57] algorithms. Both of these algorithms were introduced after the first publications and implementations of the elastic principal graph methodology (see Table 1). The SimplePPT algorithm solves the ‘reverse graph embedding’ problem by optimizing a functional which has some similarity with (7) and is exploiting to some extent the (pluri-)harmonic embedment [22]. Unlike ElPiGraph, both algorithms learn a low-dimensional layout of the graph nodes representing distances between them in the full data space, which is used for data visualization. In contrast, ElPiGraph constructs the low-dimensional principal tree layout by using external algorithms such as metro map layout [21] or weighted force-directed layout as in this this paper. Both SimplePPT and DDRTree do not exploit the principle of topological grammars, relying on estimating the principal tree structure with the Minimal Spanning Tree algorithm. However, they do test multiple principal tree structures during the learning process even if much less extensively compared to ElPiGraph. DDRTree is different from the SimplePPT in that it exploits an additional projection onto the locally optimal low-dimensional linear manifold, at each iteration, such that the structure of the principal tree is always learnt in a low-dimensional space.

In order to compare these algorithms with ElPiGraph, we used previously published LizardBrain generator of noisy branching data point clouds [56]. Briefly, it generates data points in a unit *m*-dimensional hypercube around a set of non-linear (e.g., parabolic) branches, such that each next branch starts from a randomly selected point on one of the previously generated branches in a random direction. A characteristic example of the comparison between three methods is shown in Supplementary Figure S3. In all tests, SimplePPT tends to produce the number of stars larger than the ground truth number, and their number increases with the number of graph nodes. Conversely, DDRTree underestimated the number of stars in the graph in all tests, and mixed many branches of the true graph together. ElPiGraph was able to correctly detect certain number of branching points, missing those where the initial branches were connected too close to their ends, interpreting this situation as a turn. Quite in contrast to SimplePPT, ElPiGraph produced graph structures which stabilized after inserting certain number of graph nodes and did not change further. The source code for reproducing the comparison tests is available from the Python repository of ElPiGraph.

### 3.3. Inferring Branching Pseudo-Time Cell Trajectories from Single-Cell RNASeq Data via ElPiGraph

**Figure 4:**
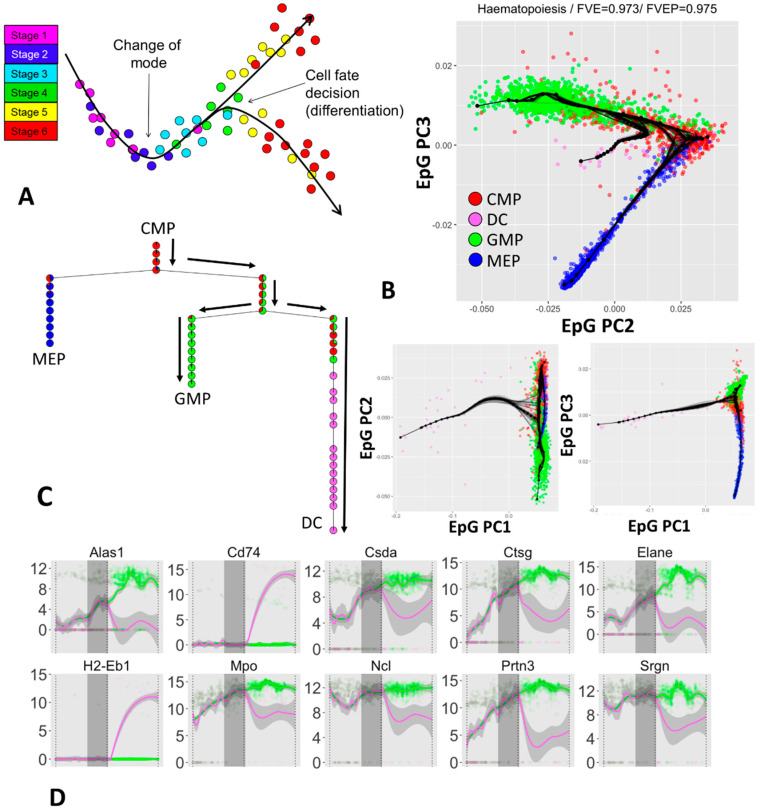
Quantification of branching cellular trajectories from single cell scRNASeq data using ElPiGraph. (**A**) Diagrammatic representation of the concept behind branching cellular trajectories and biological pseudotime in an arbitrary 2D space associated with gene expression: as cells progress from Stage 1 they differentiate (Stages 2 and 3) and branch (Stage 4) into two different subpopulations (Stages 5 and 6). Local distances between the cells indicate epigenetic similarity. Note how embedding a tree into the data allow recovering genetic changes associated with cell progression into the two differentiated states. (**B**) Application of ElPiGraph to scRNA-Seq data [59]. Each point indicates a cell and is color-coded using the cellular phenotype specified in the original paper. One hundred bootstrapped trees are represented (in black), along with the tree fitted on all the data (black nodes and edges). Projection on ElPiGraph principal components 2 and 3 is shown enlarged as the most informative. The fraction of variance of the original data explained by the projection on the nodes (FVE) and on the edges (FVEP) is reported on top of the plot. (**C**) Diagrammatic representation of the distribution of cells across the branches of the tree reconstructed by ElPiGraph with the same color scheme as in panel B. Pie charts indicate the distribution of populations associated with each node. The black arrows highlight a particular cell fate decision trajectory from CMP to GMP and DC. (**D**) Single-cell dynamics of gene expression along the path from the root of the tree (at the top of panel C) to the branch corresponding to DC commitment and GMP commitment. The expression profiles have been fitted by a LOESS smoother, colored according to the majority cell type in the branch, with a 95% confidence interval (in grey). The vertical grey area represents a 95% confidence interval obtained by projecting the relevant branching points of the resampled tree showed in panel B on the path of the principal graph.

Thanks to the emergence of single cell assays, it is now possible to measure gene expression levels across thousands to millions of single cells (scRNA-Seq data). Using these data, it is possible to look for paths in the data that may be associated with the level of cellular commitment w.r.t. a specific biological process and use the position (called pseudotime) of cells along these paths (called trajectories) to explore how gene expression reflect changes in cell states as the cells progressively commit to a given fate (Figure 4A). This kind of analysis is a powerful tool that has been used, e.g., to explore the biological changes associated with development [40], cellular differentiation [41,42,43], and cancer biology [44,58].

ElPiGraph is being used as part of STREAM [33], an integrated analysis tool that provides a set of preprocessing steps and a powerful interface to analyze scRNA-Seq or other single cell data to derive, for example, genes differentially expressed across the reconstructed paths. An extensive benchmarking of the ability of ElPiGraph in dealing with biological data as part of the STREAM pipeline is discussed elsewhere [33]. MERLOT represents another use of ElPiGraph as a part of scRNA-Seq data analysis pipeline [34]. In order to illustrate the work of ElPiGraph on real-life datasets, here we will showcase application of it to the analysis of scRNA-Seq dataset related to hematopoiesis [59], which was not published before.

Hematopoiesis is an important biological process that depends on the activity of different progenitors. To better understand this process researchers used scRNA-Seq to sequence cells across four different populations [59]: common myeloid progenitors (CMPs), granulo-monocyte progenitors (GMPs), megakaryocyte-erythroid progenitors (MEPs), and dendritic cells (DCs). Using the same preprocessing pipeline as STREAM, which includes selection of the most variant genes via a LOESS-based method [60] and dimensionality reduction by Modified Local Linear Embedment (MLLE) [61], we obtained a 3D projection of the original data and applied ElPiGraph with resampling (Figure 4B–E).

As Figure 4B,C show, ElPiGraph is able to recapitulate the differentiation of CMPs into GMPs and MEPs on the MLLE-transformed data. A further branching point corresponding to early-to-intermediate GMPs into DCs can be observed. This differentiation trajectory is characterized by a visible level of uncertainty associated with the branching point. The emerging biological picture is compatible with DC emerging at across a specific, but relatively broad, range of commitment of GMPs. However, it is worth stressing that the number of DCs present in the dataset under analysis is relatively small (30 cells), and hence that the higher uncertainty level associated with the branching from GMPs to DC may be simply due to the relatively small number of DC sequenced at an early committed state.

Notably, when we used ElPiGraph on the expression matrix restricted to the most variant genes and used PCA to retain the leading 250 components, we could still discover the branching point associated with the differentiation of CMPs into GMPs and MEPs. However, DCs do not seem to produce an additional branching, suggesting that MLLE can be an essential dimensionality reduction step.

We can further explore the epigenetic changes associated to DC differentiation by projecting cells to the closest graph edge, hence obtaining a pseudotime value that we can use to explore gene expression variation across branches and look for potentially interesting patterns. Figure 4D shows the dynamics of set of notable genes selected by their high variance, large mutual information when looking at the pseudotime ordering, or significant differences across the diverging branches. Note that in all of the plots, the pseudotime with a value of 0.5 corresponds to the DC-associated branching point and the vertical grey area indicate a 95% confidence interval computed using the position of branching points of the resampled principal graphs. The confidence interval provides a good indication of the uncertainty associated with the determination of the branching points and provides, to a first approximation, an indication of the pseudotime range when the transcriptional programs of the two cell populations start to diverge.

### 3.4. Approximating the Complex Structure of Development or Differentiation from Single Cell RNASeq Data

Recently, several large-scale experiments designed to derive single-cell snapshots of a developing embryo [37,38] or differentiating cells in an adult organism [39] have been produced. Standard algorithms connecting single cells in a reduced-dimension transcriptomic space are capable of producing informative representations of these large datasets [62]. However, such representations can be characterised by complex distributions, with areas of varying density, varying local dimensionality and excluded regions. Hence, it may be helpful to derive data cloud skeletons, which would simplify the comprehension and study of these distributions.

**Figure 5:**
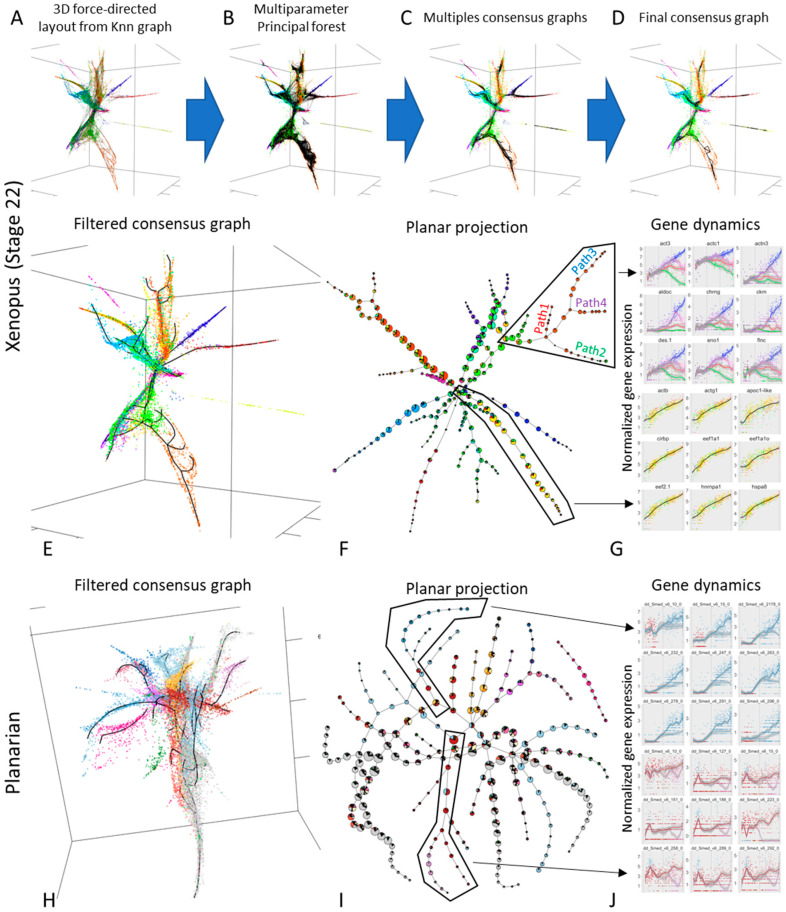
Using ElPiGraph to approximate complex datasets describing developing embryos (xenopus) or adult organisms (planarian). (**A**) A kNN graph constructed using the gene expression of 7936 cells of Stage 22 Xenopus embryo has been projected on a 3D space using a force directed layout. The color in this and the related panels indicate the population assigned to the cells by the source publication. (**B**) The coordinates of the points in panel A have been used to fit 1280 principal trees with different parameters, hence obtaining a principal forest. (**C**) The principal forest shown in panel B has been used to produce 10 consensus graphs (one for each parameter set). (**D**) A final consensus graph has been produced using the consensus graphs shown in panel C. (**E**) A final principal graph has been obtained by applying standard grammar operations to the consensus graph shown in panel D. (**F**) The associations of the different cell types to the nodes of the consensus graph shown in panel E is reported on a plane with a pie chart for each node. Note the complexity of the graph and the predominance of different cell types in different branches, as indicated the predominance of one or few colors. (**G**) The dynamics of notable genes had been derived by deriving a pseudotime for a branching structure (top) and a linear structure (bottom) present in the principal graph of panel E (see black polygons). Each point represents the gene expression of a cell and their color indicate either their associated path (top) of the cell type (bottom). The gene expression profiles have been fitted with a LOESS smoother which include a 95% confidence interval. In the top panel the smoother has been colored to highlight the different paths, with the color indicated in the text of panel F. (**H**–**J**) The same approach described by panels A–G has been used to study the single-cell transcriptome of planarians. In panel J, the color of the smoother indicates the predominant cell type on the path. Interactive versions of key panels are available at https://sysbio-curie.github.io/elpigraph/.

To this end, we used scRNA-Seq obtained from stage 22 Xenopus embryos [37] to derive a 3D force directed layout projection (Figure 5A). Given the complexity of the data, we decided to employ an advanced multistep analytical pipeline, based on ElPiGraph. As a first step, we fitted a total 1280 principal trees with 10 different trimming radiuses (to account for the differences in data density across the data space). This resulted in 10 bootstrapped principal graph ensembles (Figure 5B). Then, for each principal graph ensemble, we built a consensus graph, summarizing their features (Figure 5C). A final consensus graph was then built by combining the previously obtained ones (Figure 5D). This graph was then filtered and extended to better capture the data distribution (Figure 5E). From this analysis, clear non-trivial structures emergence: linear paths, interconnected closed loops, and branching points can be observed.

The different branches (defined as linear path between nodes of degree different from two), display a statistically significant (Chi-Squared test *p*-value < 5 × 10^−4^) associations with previously defined populations [37] (Figure 5F). Using the principal graph obtained, it is also possible to obtain a pseudotime that can be used to explore how different genes vary across the different branches (Figure 5G). Our approach was able to identify structured transcriptomic variation in a group of cells previously identified as ‘outliers’ (Figure 5G, top panel). Furthermore, our approach identified a loop in the part of the graph associated with the neural tube (Figure 5F, top left), suggesting the presence of complex diverging-converging cellular dynamics.

The same analysis pipeline was used to explore the transcriptome of the whole-organism data obtained from planarians [39]. As in the case of xenopus development data, a complex structure (Figure 5H) displaying a statistically significant association (Chi-Squared test *p*-value < 5 × 10^−4^) with previously defined cell types emerges (Figure 5I). As before, such structure can be used to identify how gene dynamics changes as cells commit to a specific cell type (Figure 5I).

## 4. Discussion

ElPiGraph represents a flexible approach for approximating a cloud of data points in a multidimensional space by reconstructing one-dimensional continuum passing through the middle of the data point cloud. This approach is similar to principal curve fitting, but allows significantly more complex topologies with, for example, branching points, self-intersections and closed paths. ElPiGraph is specifically designed to be robust with respect to the noise in the data. These features, together with an explicit control of the structural graph complexity, make ElPiGraph applicable in many scientific domains, from data analysis in molecular biology to image analysis, or the analysis of complex astronomical data.

We show that ElPiGraph scales linearly with the number of data points and dimensions and as the third power of the number of graph nodes, which can be further improved by using various heuristics outlined and partially tested in this study. This feature and also the possibility to use GPUs allows ElPiGraph to construct complex and large graphs containing up to a few thousand nodes in many dimensions. In most practical applications, the number of nodes in a principal graph is advised to be the square root of the number of data points. Therefore, ElPiGraph can be potentially applied to unravel the complexity of large and highly structured data point clouds containing millions of points.

The main challenge of approximating a dataset characterized by complex non-linear patterns with a principal graph is finding the optimal graph topology matching the underlying data structure. In order to do that, ElPiGraph starts with an initial guess of the graph topology and graph injection map, and apply a set of pre-defined graph rewriting rules, called graph grammar, in order to explore the space of admissible graph structures via a gradient descent-like algorithm by selecting the locally optimal one (Figure 1D). One of the graph grammars allows exploring the space of possible tree-like graph topologies, but changing the grammar set allows simpler or more complex graphs to be derived.

The result of ElPiGraph can be affected by data points located far from the representative part of the data point cloud. In order to deal with this, ElPiGraph exploits a trimmed approximation error which essentially makes data points located farther than the trimming radius (*R*_0_) from any graph node invisible to the optimization procedure. However, when growing, the principal graphs can gradually capture new data points, being able to learn a complex data structure starting from a simple small fragment of it. Such a ‘from local to global’ approach provides robustness and flexibility for the approximation; for example, it allows solving the problem of self-intersecting manifold clustering.

Also, the final structure of the principal graphs can be sensitive to the non-essential particularities of local configurations of data points, especially in those areas where the local intrinsic data dimensionality becomes greater than one. In order to limit the effect of a finite data sample and estimate the confidence of inferred graph features (e.g., branching points, loops), ElPiGraph applies a principal graph ensemble approach by constructing multiple principal graphs after subsampling the dataset. Posterior analysis of the principal graph ensemble allows assigning confidence scores for the topological features of the graph (i.e., branching points) and confidence intervals on their locations. The properties of principal graph ensemble can be further recapitulated into a consensus principal graph possessing much more robust properties than any of the individual graphs. In addition, ElPiGraph allows an explicit control of the graph complexity via penalizing the appearance of higher-order branching points.

It is worth stressing that efficient data approximation algorithms do not overcome the need for careful data pre-processing, selection of the most informative features, and filtering of potential artefacts present in the data; since the resulting data manifold topology crucially depends on these steps. The application of ElPiGraph to scRNA-Seq data describing hematopoiesis clearly exemplifies this aspect, as the use of MLLE was able to highlight important features of the data that were otherwise hard to distinguish.

The methodological approach employed by elastic principal graphs is not limited to reconstructing intrinsically one-dimensional data approximators. Similar to self-organizing maps, principal graphs organized into regular grids can effectively approximate data by manifolds of relatively low intrinsic dimensions (up to four dimensions in practice due to the exponential increase in the number of nodes). Previously such an approach was implemented in the method of elastic maps [2,10], which requires introducing non-primitive elastic graphs characterized by several selections of subgraphs (stars) in the elastic energy definition. The method of elastic maps has been successfully applied for non-linear data visualization, within multiple domains [11,63,64,65,66]. Conceptually, it remains an interesting research direction to explore the application of elastic principal graph framework to reconstructing intrinsic data manifolds characterized by varying intrinsic dimension.

Overall, ElPiGraph enables construction of flexible and robust approximators of large datasets characterized by topological and structural complexity. Furthermore, ElPiGraph does not rely on a specific feature selection or dimensionality reduction techniques, making it easily integrable into different pipelines, as it was recently the case for the single cell genomic data. All in all, this indicates that ElPiGraph can serve a valuable method in the information geometry toolbox for the exploration of increasingly complex and large datasets.

## Figures and Tables

**Table 1:** Elements of the principal graph methodology.

Element	Initial Publication	Principal Advances
Principal curves	Hastie and Stuelze, 1989 [24]	Definition of principal curves based on self-consistency
Piece-wise linear principal curves	Kégl et al., 1999 [25]	Length constrained principal curves, polygonal line algorithm
Elastic energy functional for elastic principal curves and manifolds	Gorban, Rossiev, Wunsch II, 1999 [26]	Fast iterative splitting algorithm to minimize the elastic energy, based on sequence of solutions of simple quadratic minimization problems
Method of elastic maps	Zinovyev, 2000 [27], Gorban and Zinovyev, 2001 [28]	Construction of principal manifold approximations possessing various topologies
Principal Oriented Points	Delicado, 2001 [29]	Principal curves passing through a set of principal oriented points
Principal graphs specialized for image skeletonization	Kégl and Krzyzak, 2002 [30]	Coining the term principal graph, an algorithm extending the polygonal line algorithm, specialized on image skeletonization
Self-assembling principal graphs	Gorban and Zinovyev, 2005 [10]	Simple principal graph algorithm, based on application of elastic map method, specialized on image skeletonization
General purpose elastic principal graphs	Gorban, Sumner, Zinovyev, 2007 [20]	Suggesting the principle of (pluri-)harmonic graph embedding, coining the terms ‘principal tree’ and ‘principal cubic complex’ with algorithms for their construction
Topological grammars	Gorban, Sumner, Zinovyev, 2007 [20]	Exploring multiple principal graph topologies via gradient descent-like search in the space of admissible structures
Explicit control of principal graph complexity	Gorban and Zinovyev, 2009 [17]	Introducing three types of principal graph complexity and ways to constrain it
Regularized principal graphs	Mao et al., 2015 [22]	Formulating reverse graph embedding problem. Suggesting SimplePPT algorithm. Further development in Mao et al., 2017 [19]
Robust principal graphs	Gorban, Mirkes, Zinovyev, 2015 [31]	Using trimmed version of the mean squared error, resulting in the ‘local’ growth of the principal graphs and robustness to background noise
Domain-specific adaptations of principal graphs	Qiu et al., 2017 [32] Chen et al., 2019 [33] Para et al., 2019 [34]	Use of principal graphs in single cell data analysis as a part of pipelines Monocle, STREAM, MERLoT. Introducing heuristics for problem-specific initializations of principal graphs. Benchmarked in Saelens et al., 2019 [35].
Partition-based graph abstraction	Wolf et al., 2019 [36]	Dealing with non-tree like topologies, large-scale and multi-scale data analysis using graphs
Excessive branching control	This publication	Introducing a penalty for excessive branching of elastic principal graphs (α parameter)
Principal graph ensemble approach	This publication	Estimating confidence of branching point positions, constructing consensus principal graphs
Reducing the computational complexity of elastic principal graphs	This publication	Accelerated procedures and heuristics in order to enable constructing large principal graphs
Use of GPUs for constructing elastic principal graphs	This publication	Scalable Python and R implementations of elastic principal graphs (ElPiGraph), improved scalability and introducing various plotting functions

List of used abbreviations: GDA—geometrical data analysis; TDA—topological data analysis; ElPiGraph—elastic principal graphs; GPU—graphical processing unit; CPU—central processing unit; kNN—k neareast neighbors; SOM—self-organizing maps; LLE—local linear embedding; UMAP—Uniform Manifold Approximation and Projection for Dimension Reduction.

## Supplementary Materials

The following are available online at: https://www.mdpi.com/1099-4300/22/3/296/s1.
