# Supplementary material for: Robust and Scalable Learning of Complex Intrinsic Dataset Geometry via ElPiGraph
Source: Entropy (Basel). 2020 Mar 4;22(3):296. doi: 10.3390/e22030296 (PMC7516753; doi:10.3390/e22030296)
Supplement: Supplementary file 1 [file entropy-22-00296-s001.pdf]

entropy

MDPI

Supplementary Figures for the manuscript 'Robust and Scalable Learning of Complex Intrinsic Dataset Geometry via ElPiGraph' by Albergante et al.

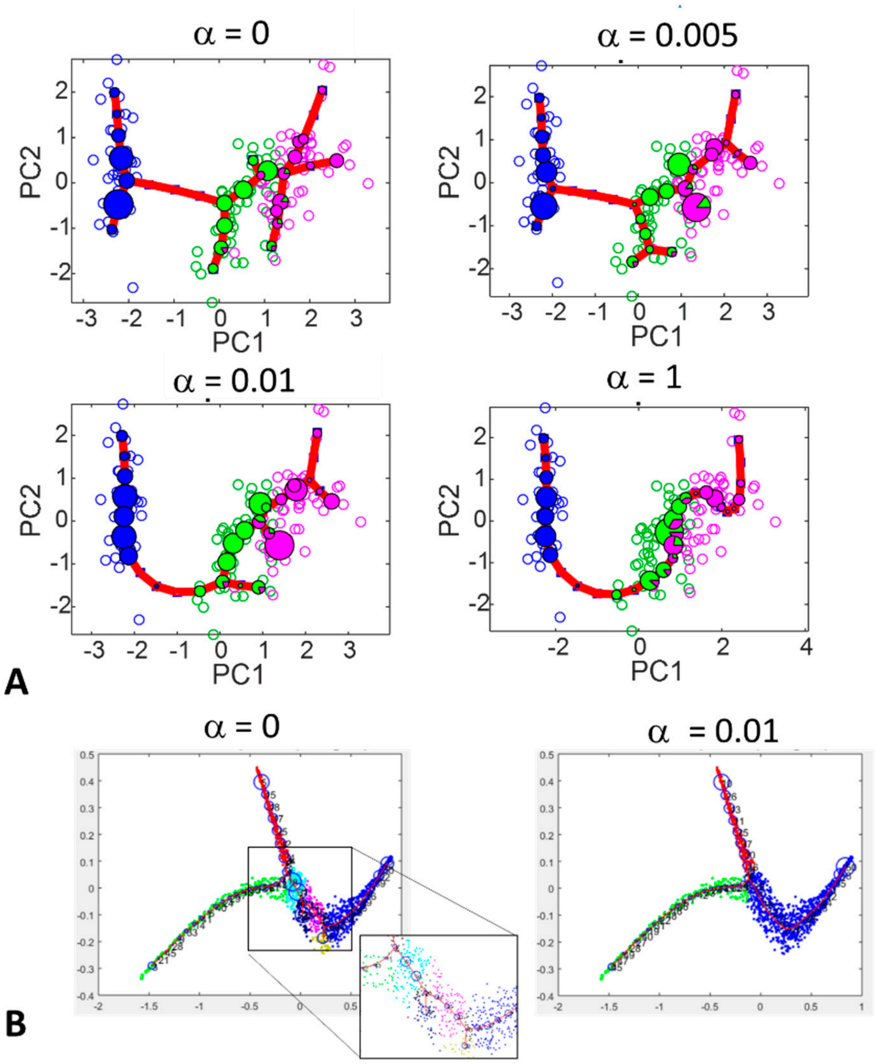

Figure S1. Explicit control for topological complexity in ElPiGraph, using α parameter. (**A**) Iris dataset, approximated by ElPiGraph with default parameters, using increasing values of α. Here, the color of the points designates the point class labels. Each node of the graph represented as a pie-chart, which size is proportional to the number of data points projected into it, and the size of the sectors are proportional to the number of data points of certain class. (**B**) Synthetic dataset characterized by a 'thick turn' pattern (when the local variance of the dataset increases in the region characterized by the largest curvature of the principal curve). Using explicit control for topological complexity, it is possible to suppress the small branches while retaining the major one. Small fictitious branches appear here due to effective increase of local data dimension, which does not change the underlying data topology. Here, the data point color visualizes the partitioning of the data by principal node branches: each color corresponds to the data points projected on a particular branch of the tree. The nodes of the graph are shown by empty circles which size is proportional to the number of data points projected into it.

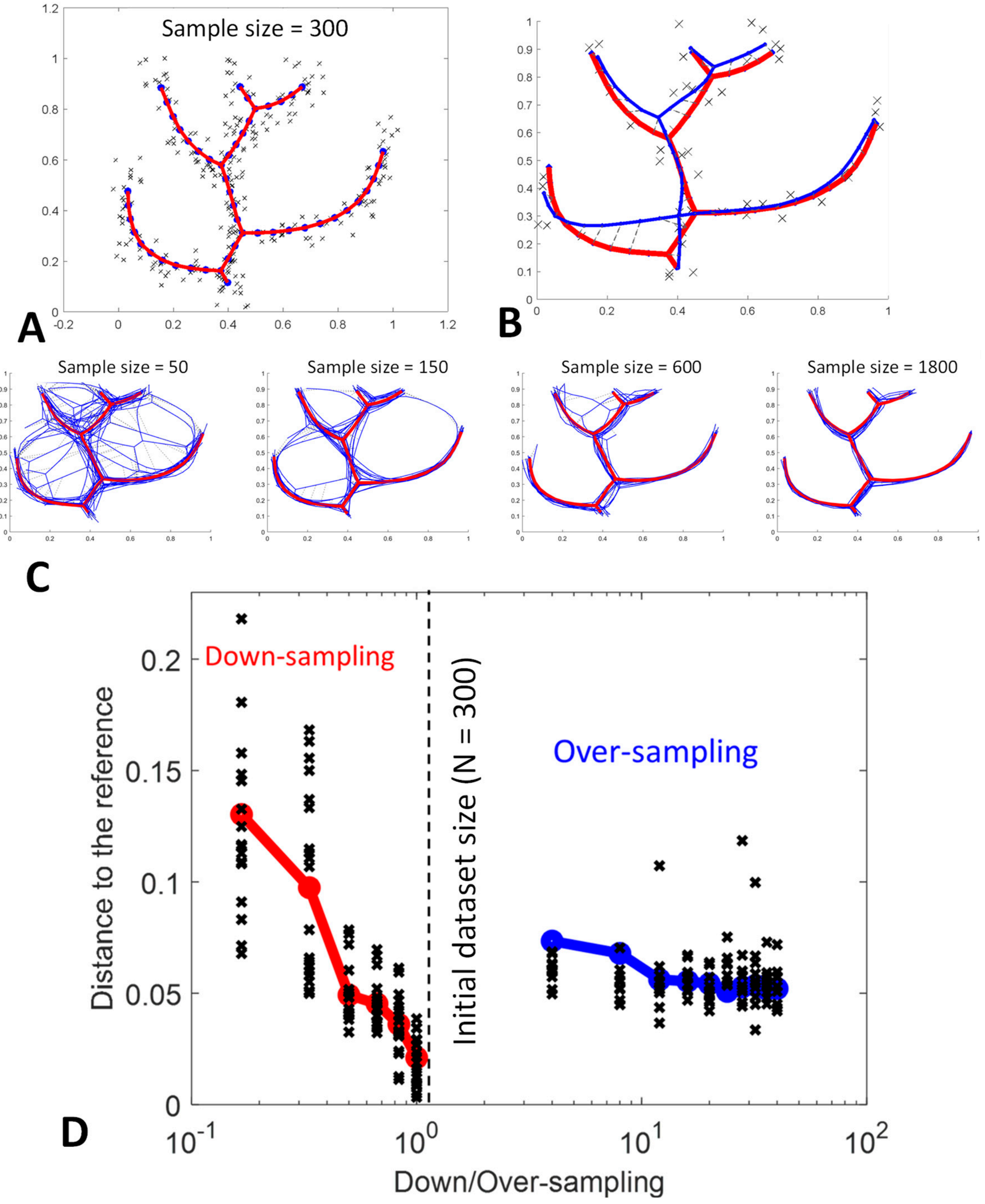

Figure S2. Effect of downsampling or over-sampling on the robustness of the construction of the principal tree in a simple 2D branching data example. (**A**) Initial dataset and the principal tree reconstructed (ElPiGraph was run with parameters λ=0.02, μ=0.2, α=0.005 and s = 50 nodes). (**B**) Principle of computing the distance between the reference tree (shown in red) and a tree constructed on a subsample of data (shown by crosses). For each node of the subsample principal tree we perform the nearest neighbor search of the reference tree node. The distance is computed as ratio between the sum of distances between all nodes matched in the reference and the subsample tree (shown by dashed grey lines) and the total length of the reference tree (sum of all edge lengths). (**C**) Runs of ElPiGraph on 20 subsamples of the initial dataset of different sizes. (**D**) Dependence of the distance

from a subsample tree to the reference tree (y-axis), based on 20 random subsamples of different sizes. The x-axis is a fraction of the sample with respect to the initial dataset.

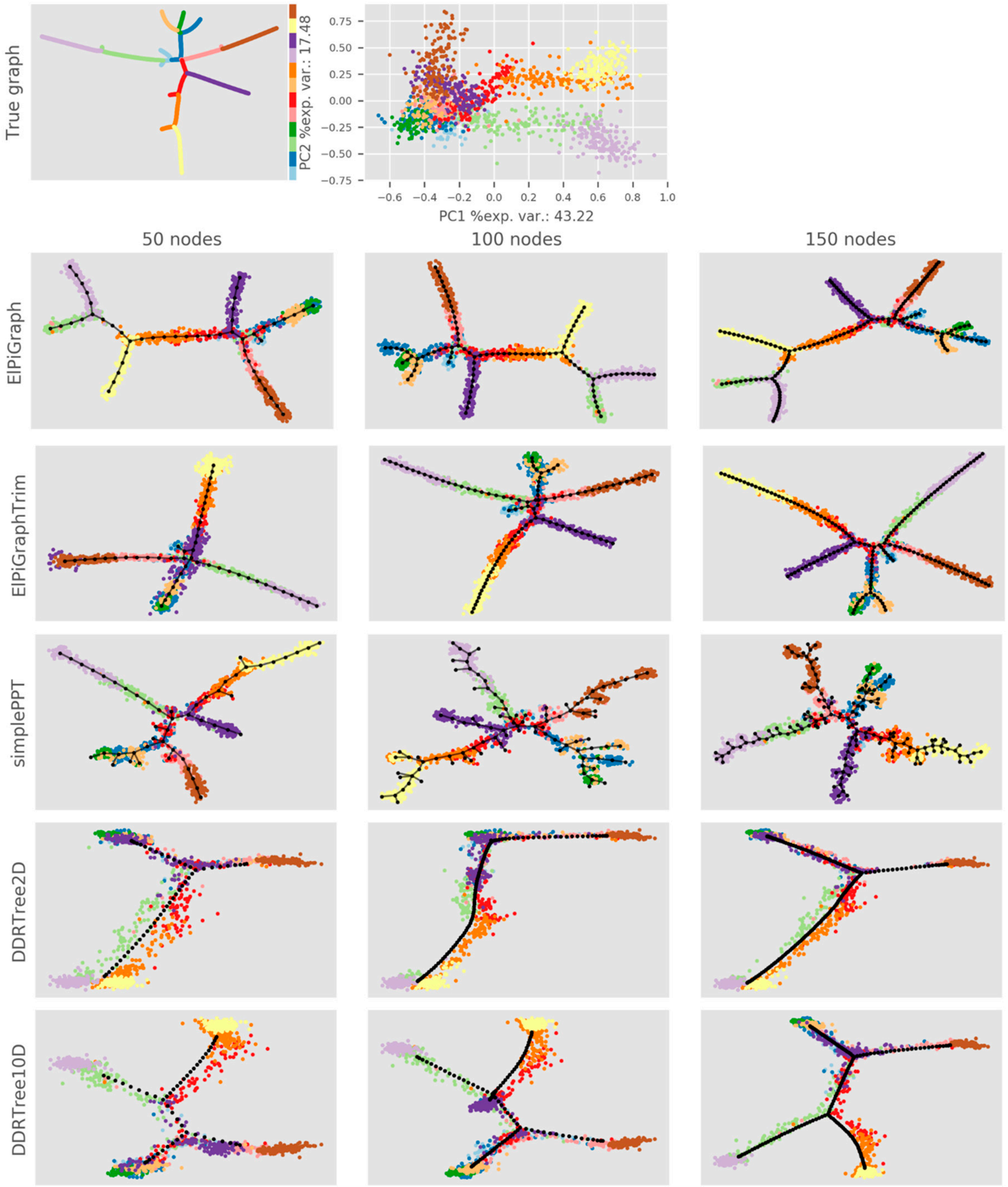

Figure S3. Comparison of ElPiGraph with SimplePPT and DDRTree methods, using generator of synthetic branching data distributions LizardBrain (see the main text for description). **First row, left**: The underlying topology of an example generative model, consisting of 12 non-linear branches embedded in the 10-dimensional unit hypercube. The structure has 11 stars. **First row, right**: Projection of the generated noisy dataset on the plane of the first two principal components. The three algorithms are run using 50, 100, 150 graph nodes.
ElPiGraph row: principal graph reconstructed from unlabeled data with default parameters (from the simplest two-node initial guess). The graph contains 5, 7, and 8 stars at three learning steps. Note that the green and violet branches were incorrectly wired with the orange and yellow branches early in the learning process. **ElPiGraphTrim row**: robust principal graph constructed using TrimmingRadius=0.3 option. The algorithm detected 5 stars out of 11, missing those connections when the initial branches were connected close to their ends. **SimplePPT row**: SimplePPT algorithm applied with default parameters. **DDRTree2D row**: DDRTree algorithm

applied with default parameters, using two-dimensional projection on the local linear manifold at each iteration. **DDRTree10D row**: DDRTree algorithm applied with default parameters except for the linear manifold dimension set to 10. ElPiGraph and simplePPT plots were produced by applying the Kamada–Kawai force-directed layout algorithm and placing data points next to their closest edge. For DDRTree we do not show a force-directed layout but directly the output data and node embedding. The implementation of DDRTree provided by Cole Trapnell laboratory has a known issue and does not return the adjacency matrix of the graph, preventing us from generating a force-directed layout (https://github.com/cole-trapnell-lab/DDRTree/issues/1). The implementation of simplePPT was taken from VISION: https://rdrr.io/github/YosefLab/VISION/ https://rdrr.io/github/YosefLab/VISION/man/applySimplePPT.html. The implementation of DDRTree was taken from MONOCLE 3 alpha installation instructions: http://cole-trapnell-lab.github.io/monocle-release/monocle3/ which refers to: https://github.com/cole-trapnell-lab/DDRTree. The Jupyter notebook producing this graph can be found in the ElPiGraph Python repository.
